# Clinical manifestations and biomarkers to predict mortality risk in adults with invasive *Streptococcus dysgalactiae* subsp. *equisimilis* infections

**DOI:** 10.1007/s10096-024-04861-4

**Published:** 2024-06-07

**Authors:** Shigeo Hanada, Takeaki Wajima, Misako Takata, Miyuki Morozumi, Michi Shoji, Satoshi Iwata, Kimiko Ubukata

**Affiliations:** 1 Department of Respiratory Medicine, Respiratory Center, Toranomon Hospital, 2-2-2 Toranomon, Minato-ku, Tokyo, 105-8470 Japan; 2 Okinaka Memorial Institute for Medical Research, Tokyo, Japan; 3 Department of Microbiology, Tokyo Medical University, Tokyo, Japan; 4 Department of Microbiology, Faculty of Pharmacy, Meijo University, Nagoya, Japan; 5 Department of Infectious Disease, National Sanatorium Tamazenshoen, Tokyo, Japan; 6 Center for General Medicine Education, Keio University School of Medicine, Tokyo, Japan

**Keywords:** Biomarker, Risk factor for death, Invasive infection, *emm* type, *Streptococcus dysgalactiae* subsp. *equisimilis*

## Abstract

**Purpose:**

The incidence of invasive *Streptococcus dysgalactiae* subsp. *equisimilis* (iSDSE) infections is increasing in developed countries, but studies on the risk factors for death in iSDSE infections are scant. Here, we aimed to clarify risk factors and predictors of mortality in adults with iSDSE infections.

**Methods:**

A multicentre observational study of adults with iSDSE infections was conducted to investigate the effects of host factors, disease severity, biomarkers, and antibiotic regimens, and bacterial factors on 28-day mortality.

**Results:**

The overall mortality rate of 588 patients was 10.4%, with a significant increase in those aged ≥ 60 years. Most of the patients (97.4%) had underlying diseases. The mortality rate (70.4%) of patients with severe disease was significantly higher than that of patients with mild-to-moderate disease (4.3%; *p* < 0.001). The risk factors for death identified using multivariable analysis were age ≥ 60 years (hazard ratio [HR], 3.4; 95% confidence interval [CI], 1.0–11.3, *p* = 0.042); severe disease (HR, 15.0; 95% CI 7.7–29.2, *p* < 0.001); bacteraemia without primary focus (HR, 20.5; 95% CI 2.8–152.3, *p* = 0.003); serum creatinine ≥ 2.0 mg/dL (HR, 2.2; 95% CI 1.2–4.0, *p* = 0.010); serum creatine kinase ≥ 300 IU/L (HR, 2.1; 95% CI 1.1–3.8, *p* = 0.019); and macrolide resistance (HR, 1.8; 95% CI 1.0–3.3, *p* = 0.048). Treatment regimens and *emm* types were not associated with poor outcomes.

**Conclusion:**

Evaluation of clinical manifestations and biomarkers on admission is important to predict invasive SDSE infection prognosis.

**Supplementary Information:**

The online version contains supplementary material available at 10.1007/s10096-024-04861-4.

## Introduction

*Streptococcus dysgalactiae* subspecies *equisimilis* (SDSE) was proposed as a new subspecies in 1996 [1]. SDSE isolated from humans as a commensal microorganism possesses antigens belonging to Lancefield group G, C, or A [2]. Although SDSE forms relatively large glossy colonies on blood agar that are strongly β-haemolytic, several biochemical property tests, such as the pyrrolidonyl arylamidase test, are required to distinguish SDSE from other haemolytic streptococci. Moreover, SDSE had been considered substantially less pathogenic than *Streptococcus pyogenes* (GAS). However, whole-genome sequencing has revealed that SDSE possesses several virulence factors. These factors include M protein (*emm*), streptokinase, C5a peptidase, hyaluronidase, and others involved in immune evasion, as demonstrated in GAS, and systemic toxicity of streptolysin O and streptolysin S [3, 4]. Thus, SDSE and GAS are considered closely related phylogenetically and may have originated from a common precursor [5].

SDSE was considered an asymptomatic coloniser of the human upper airway, skin, and gastrointestinal tract. Recently, it has been recognised as a clinically relevant pathogen that causes a broad range of diseases, from milder illnesses such as local skin and soft tissue infections to life-threatening conditions such as streptococcal toxic shock syndrome (STSS) and necrotising fasciitis (NF), similar to those caused by GAS [6–12]. Invasive SDSE (iSDSE) infections are more common in older adults [12, 13] and individuals with underlying chronic diseases [7–10, 14, 15]. Consequently, the impact of disease burden associated with SDSE infections will grow in developed countries, which are aging rapidly.

Although studies have compared the onset age [16, 17], underlying diseases [7, 14–16], biomarkers [12], and mortality rates of iSDSE [7–9, 12, 14] with invasive GAS and invasive Group B streptococcal infections, only a few have clarified the risk factors for death in patients with iSDSE [17]. A common problem exists in that clinicians have limited awareness of the pathogenesis of SDSE. Therefore, we aimed to consolidate clinically relevant information on SDSE infections. We thus conducted this large-scale surveillance study of iSDSE infection in Japanese adults to evaluate the relative contribution of host factors, clinical manifestations, biomarkers, and bacterial factors to the 28-day mortality after admission.

## Materials and methods

### Study design

This retrospective cohort study involved a nationwide surveillance of iSDSE infections conducted from April 2010 to March 2016. One-hundred and thirty-six general hospitals with clinical microbiology laboratories throughout Japan participated in this study (Supplementary Fig. 1). During the study period, we received 686 strains derived from patients with iSDSE from the medical institutions. Ninety-eight patients (14.3%) were excluded from this study because of missing data (lack of basic information or loss on follow-up), and 588 (85.7%) cases were included for analysis.

### SDSE isolates and patient definition

We identified patients aged ≥ 18 years with iSDSE infections. iSDSE infection was defined as the isolation of SDSE from normally sterile clinical samples such as blood, cerebrospinal fluid, joint/synovial fluid, and closed pus. Strains submitted to our laboratory as SDSE from each institution were confirmed to belong to Lancefield Group A, C, or G using an agglutination test with latex agglutination reagent and re-identified according to the clinical microbiology manual. The Lancefield agglutination test results were 85.3% for group G, 12.8% for group C, and 1.9% for group A (*emm* type, *stG485* and *stG245*), respectively. Eighty-six strains that agglutinated in group A or group C were confirmed to be SDSE by 16S rRNA sequencing.

Underlying chronic disease was defined as the presence of diabetes mellitus, cardiovascular disease, active cancer treated with anti-cancer therapy, kidney disease (severe or moderate reduction in glomerular filtration rate [GFR categories G4-5, or G3; KDIGO classification]), immunocompromised conditions, including rheumatoid arthritis, systemic lupus erythematosus, asplenia, congenital immunodeficiency, haematological diseases, and high dosage of medication such as systemic corticosteroids (daily dose of prednisolone equivalent to ≥ 10 mg/day for > 2 weeks) and biologics, liver disease, artificial joint replacement, and others. Disease severity was assessed using the diagnostic criteria of the guidelines for each disease [18–21] on admission.

### Skin and soft tissue infections

The diagnostic criteria for severity assessment of skin and soft tissue infections (SSTI) are as follows [18]. For purulent SSTI, purulent skin and soft tissue infections (SSTIs; abscess); for mild infection, incision, and drainage are indicated; for moderate infection, purulent infection with systemic signs of infection; for severe infection, incision and drainage plus oral antibiotics failed, and systemic signs of infection such as a temperature > 38 °C, tachycardia (heart rate > 90 beats per minute), tachypnoea (respiratory rate > 24 breaths per minute), or abnormal white blood cell count (> 12,000 or < 4000 cells/µL); or immunocompromised status. For non-purulent SSTIs, necrotising infection, cellulitis, or erysipelas; For mild infection, typical cellulitis or erysipelas with no focus of purulence; for moderate infection, typical cellulitis or erysipelas with systemic signs of infection; for severe infection, oral antibiotic treatment failed; systemic signs of infection (as defined above under purulent infection); immunocompromised status; or clinical signs of deeper infection such as bullae, skin sloughing, hypotension, or evidence of organ dysfunction.

### Community-acquired pneumonia

The IDSA/ATS severity criteria [20] define severe community-acquired pneumonia as patients with either one of the following major criteria or three or more of the following minor criteria. Minor criteria are respiratory rate > 30 breaths/min, PaO_2_/FiO_2_ ≤ 250, multilobular infiltrates, confusion/disorientation, uraemia (blood urea nitrogen level > 20 mg/dL), leukopenia (white blood cell count < 4,000 cells/µL), thrombocytopenia (platelet count < 100,000/µL), hypothermia (core temperature < 36 °C), and hypotension requiring aggressive fluid resuscitation. Major criteria are septic shock with need of vasopressors and respiratory failure requiring mechanical ventilation.

### Bacteraemia without primary focus and other invasive SDSE infections

The surviving sepsis campaign international guidelines [21] were used for evaluating bacteraemia without primary focus and other invasive SDSE infections. For mild sepsis, a life-threatening condition caused by a dysregulated host response to infection, resulting in organ dysfunction. The criteria for septic shock were circulatory, cellular, and metabolic abnormalities in patients with sepsis, presenting as fluid-refractory hypotension requiring vasopressor therapy with associated tissue hypoperfusion (lactate level > 2 mmol/L).

### Antimicrobial susceptibility and *emm* typing

Antibiotic treatment regimens were divided into five categories: penicillin, first- or second-generation cephalosporins, third-generation cephalosporins, carbapenems, and others. SDSE susceptibility to antimicrobial agents was determined using the agar-dilution method. We also analysed the *pbp1a*, *pbp2x*, and *pbp2b* genes, which encode penicillin-binding proteins associated with- β-lactam resistance, using DNA sequencing for each *pbp* gene. In parallel, three genes that mediate macrolide- and clindamycin resistance – *mefA*, *ermA*, and *ermB* – were identified by DNA amplification [22].

Typing of *emm* encoding the M protein was performed using polymerase chain reaction (PCR). After sequencing the amplified DNA fragments, the Centers for Disease Control and Prevention (CDC) *emm* sequence database was used to identify the *emm* type (https://www.cdc.gov/vaccines/biotech/strepblast. asp).

### Statistical analysis

Fatalities occurring within 28 days of admission were defined as deaths caused by or related to iSDSE infections. Categorical variables were tested using the χ^2^ test or Fisher’s exact test. Continuous variables were compared using the Mann-Whitney U test. Cutoffs for laboratory values were determined by the distribution of each biomarker. Relationships among death, patient attributes, and biomarkers were evaluated using logistic regression analysis to obtain odds ratios (OR), 95% confidence intervals (95%CI), and p-values. Examined biomarkers were analysed as binary data after obtaining their cutoff values in advance using receiver operating characteristic (ROC) analysis. We also plotted the survival curve using Kaplan–Meier method and determined associations of disease severity, white blood cell (WBC) count, and other biomarkers with death. After assessing the presence of interaction and multicollinearity between variables, multivariable analysis, including multiple variables, was performed using the Cox proportional hazards model. Biomarkers or other variables with high correlation coefficients (> 0.5) were excluded from the multivariable analysis. Statistical significance was defined as *p* < 0.05 (two-tailed test). All analyses were performed using SPSS version 26 (IBM Corporation, Armonk, NY, USA).

## Results

### Age distribution and outcome of patients with iSDSE infection

**Fig. 1:**
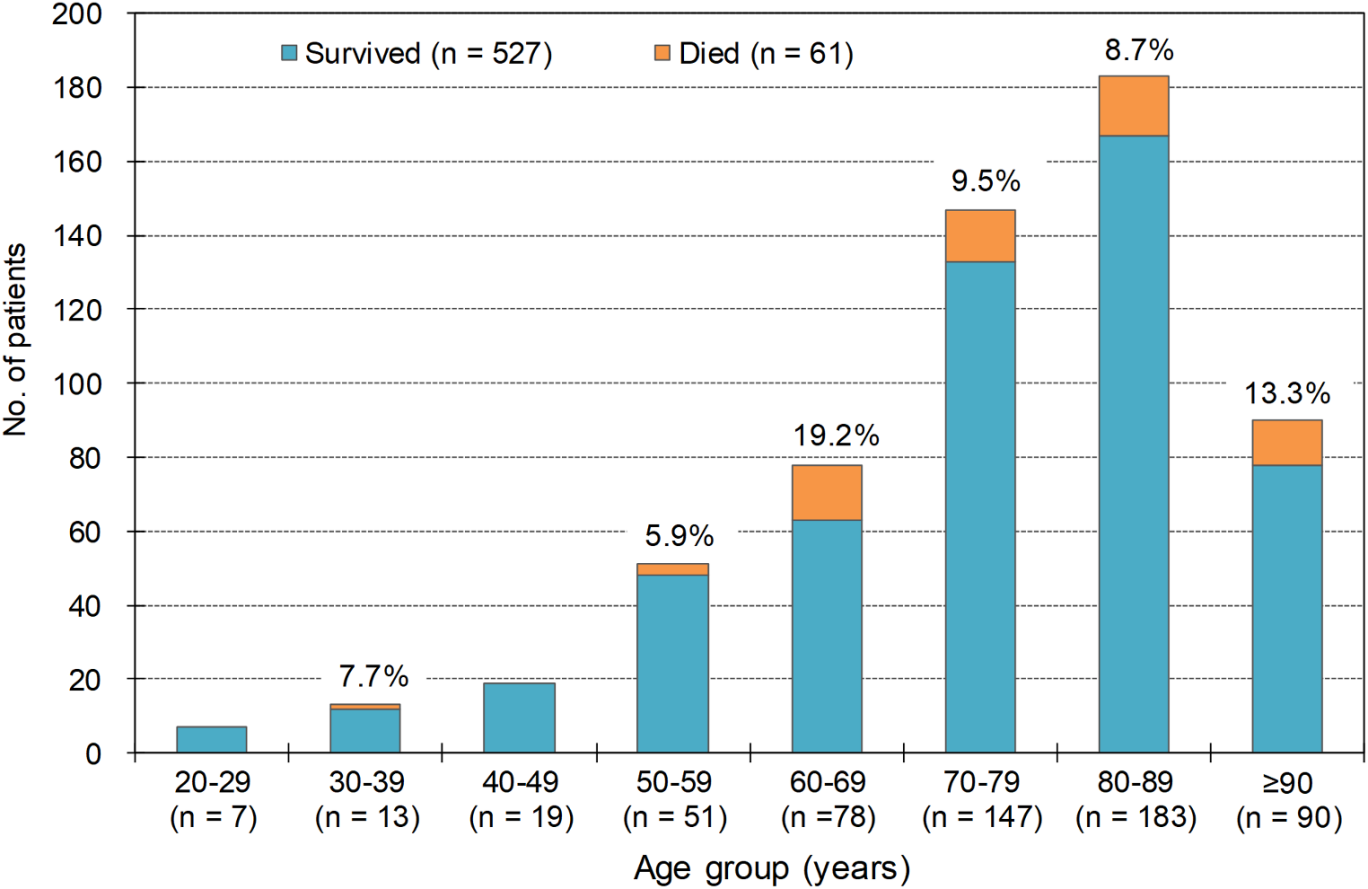
Age distribution and outcome of adult patients with invasive *Streptococcus dysgalactiae* subsp. *equisimilis* infections. Median age, 78 years (IQR 68─86); percentage above each bar, mortality rates; mortality rate in all cases, 10.4%

Three hundred nineteen cases (54.3%) were males and 269 cases (45.7%) were females. The median age of patients with iSDSE infections (*n* = 588) was 78 [interquartile range (IQR): 68─86] years. The mortality rate of patients under the age of 60 years ranged from 0.0 to 7.7%, whereas that of patients aged 60 years and older increased from 8.7 to 19.2% (see Fig.  1).

### Clinical characteristics associated with outcome

The clinical characteristics associated with outcome are shown in Table 1. Regarding disease severity, 90.8% (*n* = 534) of all patients had mild-to-moderate disease, and the mortality rate was 4.3% (*n* = 23). The remaining 9.2% of patients (*n* = 54) identified as having severe disease had a significantly increased mortality rate of 70.4% (*n* = 38; *p* < 0.001). Of the 491 patients in whom the presence or absence of underlying diseases could be clarified, 97.4% (*n* = 478) had underlying diseases. However, there were no underlying diseases that showed a significant difference between the groups. Bacteraemia without primary focus and cellulitis were the most common manifestations, accounting for 36.9% (*n* = 217) and 35.2% (*n* = 207) of all patients, respectively. Significant differences associated with fatal outcome were obtained in patients with bacteraemia without primary focus, NF, meningitis, and STSS.

**Table 1:** Characteristics of adult patients with invasive *Streptococcus dysgalactiae* subsp. *equisimilis* infections associated with fatal outcome

Characteristic	Overall	Survived (*n* = 527)	Died (*n* = 61)	*P*-value	Univariate analysis OR (95% CI)
**Age (years)**					
< 60	90	86 (95.6)	4 (4.4)	Ref	
60‒69	78	63 (80.8)	15 (19.2)	**0.005**	**5.1 (1.6‒16.2)**
70‒79	147	133 (90.5)	14 (9.5)	0.162	2.3 (0.7‒7.1)
≥ 80	273	245 (89.7)	28 (10.3)	0.102	2.5 (0.8‒7.2)
**Gender**					
Male	319	281 (88.1)	38 (11.9)	Ref	
Female	269	246 (91.4)	23 (9.6)	0.185	0.7 (0.4‒1.2)
**Disease Severity** ^a^					
Mild-to-moderate	534	511 (95.7)	23 (4.3)	Ref	
Severe	54	16 (29.6)	38 (70.4)	**< 0.001**	**52.8 (25.7‒108.2)**
**Underlying diseases**					
**With any underlying diseases and conditions** ^b^	478	427 (89.3)	51 (10.7)		
Diabetes mellitus	145	132 (91.0)	13 (9.0)	0.505	0.8 (0.4‒1.5)
Cardiovascular disease	132	116 (87.9)	16 (12.1)	0.446	1.3 (0.7‒2.4)
Malignancy	104	94 (90.3)	10 (9.6)	0.772	0.9 (0.4‒1.9)
Kidney disease	67	56 (83.6)	11 (16.4)	0.086	1.9 (0.9‒3.9)
Immunocompromised ^c^	36	33 (91.7)	3 (8.3)	1.000	0.8 (0.2‒2.6)
Liver disease ^d^	35	31 (88.6)	4 (11.4)	0.834	1.1 (0.4‒3.3)
Artificial joint replacement	6	5 (83.3)	1 (16.7)	0.484	1.7 (0.2‒15.2)
Others ^e^	50	42 (84.0)	8 (16.0)	0.240	1.6 (0.7‒3.7)
**Clinical manifestations**					
Bacteraemia without primary focus	217	178 (82.0)	39 (18.0)	**< 0.001**	**3.5 (2.0‒6.0)**
Cellulitis	207	206 (99.5)	1 (0.5)	**< 0.001**	**0.0 (0.0‒0.2)**
Septic arthritis	46	45 (97.8)	1 (2.2)	0.091	0.2 (0.0‒1.3)
Bacteraemic pneumonia	31	25 (80.6)	6 (19.4)	0.100	2.2 (0.9‒5.6)
Abscess except for skin	23	22 (95.7)	1 (4.3)	0.352	0.4 (0.1‒2.9)
Necrotising fasciitis	12	7 (58.3)	5 (41.7)	**0.002**	**6.6 (2.0‒21.6)**
Osteomyelitis/spondylitis	12	11 (91.7)	1 (8.3)	0.815	0.8 (0.1‒6.2)
Infective endocarditis	9	9 (100)	0	‒	‒
Erysipelas	8	8 (100)	0	‒	‒
Meningitis	5	2 (40.0)	3 (60.0)	**0.005**	**13.6 (2.2‒82.9)**
Toxic shock syndrome	4	1 (25.0)	3 (75.0)	**0.005**	**27.2 (2.8‒265.8)**
Others ^f^	14	13 (92.9)	1 (7.1)	0.690	0.7 (0.1‒5.1)
**Antibiotic used initially** ^g^					
Penicillin	160	148 (92.5)	12 (7.5)	Ref	
1st or 2nd cephalosporins ^h^	143	134 (93.7)	9 (6.3)	0.680	0.8 (0.3‒2.0)
3rd cephalosporins	150	135 (90.0)	15 (10.0	0.437	1.4 (0.6‒3.0)
Carbapenems	76	60 (78.9)	16 (21.1)	**0.004**	**3.3 (1.5‒7.4)**
Others ^i^	33	29 (87.9)	4 (12.1)	0.385	1.7 (0.5‒5.6)
**Macrolide susceptibility**					
Susceptible	477	437 (91.6)	40 (8.4)	Ref	
Resistance ^j^	111	90 (81.1)	21 (18.9)	**0.001**	**2.5 (1.4‒4.5)**
***emm *** **type**					
*stG6792*	161	144 (89.4)	17 (10.6)	Ref	
Others	427	383 (89.7)	44 (10.3)	0.928	1.0 (0.5‒1.8)

^a^ Disease severity was assessed using the diagnostic criteria of the guidelines for each disease [19–22]

^b^ The presence or absence of an underlying disease could be determined in 491 cases, excluding 97 cases that were unknown. Of these, 478 (97.4%) cases had underlying diseases. Each underlying disease includes overlapping cases

^c^ Includes rheumatoid arthritis (*n* = 6), systemic lupus erythematosus (*n* = 4), asplenia, congenital immunodeficiency, haematological diseases, and high dosage of medications such as systemic corticosteroids [daily dose of prednisolone equivalent to ≥ 10 mg/day for > 2 weeks) and biologics]

^d^ Includes chronic hepatitis or cirrhosis secondary to alcohol abuse or viral infection

^e^ Includes cerebrovascular disease (*n* = 13), Parkinson’s disease (*n* = 10), deep venous thrombosis (*n* = 3), chronic obstructive pulmonary disease (*n* = 3), myasthenia gravis (*n* = 3), hypothyroidism (*n* = 2), and others (*n* = 16)

^f^ Includes cholangitis (*n* = 4), peritonitis (*n* = 2), and endophthalmitis, etc

^g^ Five-hundred and sixty-two cases, excluding 26 unknown cases. These numbers exclude duplicate counts in severe patients treated with combination therapy

^h^ 1st, 2nd, and 3rd refer to 1st, 2nd, and 3rd generation cephalosporins

^i^ Including clindamycin (*n* = 28), vancomycin (*n* = 19), quinolones (*n* = 11), teicoplanin (*n* = 5), and gentamicin (*n* = 4). These numbers contain duplicate counts in severe patients treated with combination therapy. There were no patients treated with macrolides in either group

^j^ Possesses *mefA* (*n* = 9), *ermA* (*n* = 49), or *ermB* (*n* = 53) genes that mediate macrolides resistance. Strains possessing the *mefA* gene are susceptible to clindamycin, and strains possessing *ermA* or *ermB* genes are resistant to clindamycin

The categories of antimicrobial agents administered as initial treatment did not differ between the groups, except for carbapenems, which were mainly used in the mortality group (Supplementary Fig. 2). For macrolides and clindamycin agents, 18.9% of isolates possessed *ermA*, *ermB*, or *mefA*, and there were significantly more strains with resistance genes in the mortality group. There was no significant difference in mortality between *stG6792*, which was the most predominant *emm* type, and the other types.

### Association of laboratory findings with outcome

Based on the cut-off value of each biomarker, the significant difference, hazard ratio (HR), and 95% confidence interval (CI) between the survival and mortality groups were calculated (Table 2). Patients with a WBC count < 4 × 10^3^ cells/µL or a platelet (PLT) count < 10 × 10^4^ cells/µL on admission showed a significant difference with high HRs in the mortality group. The C-Reactive protein level (CRP, ≥ 20 mg/dL) also significantly differed, but with low HRs. Abnormal levels of aspartate aminotransferase (AST, ≥ 100 IU/L), blood urea nitrogen (BUN, ≥ 30 mg/dL), serum creatinine (Cr, ≥ 2.0 mg/dL), serum creatine kinase (CK, ≥ 300 IU/L), and lactate dehydrogenase (LDH, ≥ 300 IU/L) were higher in the mortality group than in the survival group (*p* < 0.001). The HRs of these biomarkers ranged from 1.8 for alanine aminotransferase (ALT) to 10.1 for WBC counts. The ROC curves of biomarkers are provided in Supplementary Fig. 3.

**Table 2:** Laboratory findings associated with outcomes in adults with invasive *Streptococcus dysgalactiae* subsp. *equisimilis* infections

Biomarker (cut-off value) ^a^	Survived (*n* = 527)		Died (*n* = 61)			
	No. of assessed	Median (IQR^b^) *n* (%)	No. of assessed	Median (IQR^b^) *n* (%)	*P*-value	HR (95% CI) ^c^
WBC (x 10^3^ cells/µL)	525	12.2 (8.6‒15.7)	60	6.2 (2.8‒12.6)		
(< 4 × 10^3^ cells/µL)		25 (4.8)		24 (40.0)	**< 0.001**	**10.1 (6.0‒16.9)**
PLT (x 10^4^ cells/µL)	524	17.1 (12.8‒22.0)	60	11.4 (5.9‒16.0)		
(< 10 × 10^4^ cells/µL)		62 (11.8)		27 (45.0)	**< 0.001**	**5.3 (3.2‒8.8)**
CRP (mg/dL)	521	8.4 (2.3‒17.2)	59	16.0 (7.8‒25.6)		
(≥ 20 mg/dL)		96 (18.4)		21 (35.6)	**0.002**	**2.3 (1.3‒3.9)**
AST (IU/L)	524	26.0 (19.8‒43.0)	60	65 (34.8‒114.3)		
(≥ 100 IU/L)		39 (7.4)		19 (31.7)	**< 0.001**	**5.0 (2.9‒8.6)**
ALT (IU/L)	522	18.0 (12.0‒29.0)	60	26.5 (18.5‒61.3)		
(≥ 100 IU/L)		24 (4.6)		5 (8.3)	0.199	1.8 (0.7‒4.6)
BUN (mg/dL)	522	21.0 (14.8‒30.6)	56	48.0 (28.2‒62.3)		
(≥ 30 mg/dL)		144 (27.6)		41 (73.2)	**< 0.001**	**6.4 (3.5‒11.6)**
Cr (mg/dL)	522	0.9 (0.7‒1.2)	58	1.8 (1.1‒2.7)		
(≥ 2.0 mg/dL)		66 (12.6)		27 (46.6)	**< 0.001**	**5.1 (3.0‒8.6)**
CK (IU/L)	460	101 (58‒230)	54	379 (170‒1224)		
(≥ 300 IU/L)		98 (21.3)		32 (59.3)	**< 0.001**	**4.8 (2.8‒8.2)**
LDH (IU/L)	502	242 (200‒314)	59	346 (259‒560)		
(≥ 300 IU/L)		149 (29.7)		39 (66.1)	**< 0.001**	**4.2 (2.4‒7.2)**

^a^*Abbreviations *WBC, white blood cell; PLT, platelet; CRP, C-reactive protein; AST, aspartate aminotransferase; ALT, alanine aminotransferase; BUN, blood urea nitrogen; Cr, creatinine; CK, creatine kinase; LDH, lactate dehydrogenase

^b^ IQR: interquartile range

^c^ HR: hazard ratio, 95% CI: confidence interval

### Disease severity and death in relation to the time elapsed after admission

**Fig. 2:**
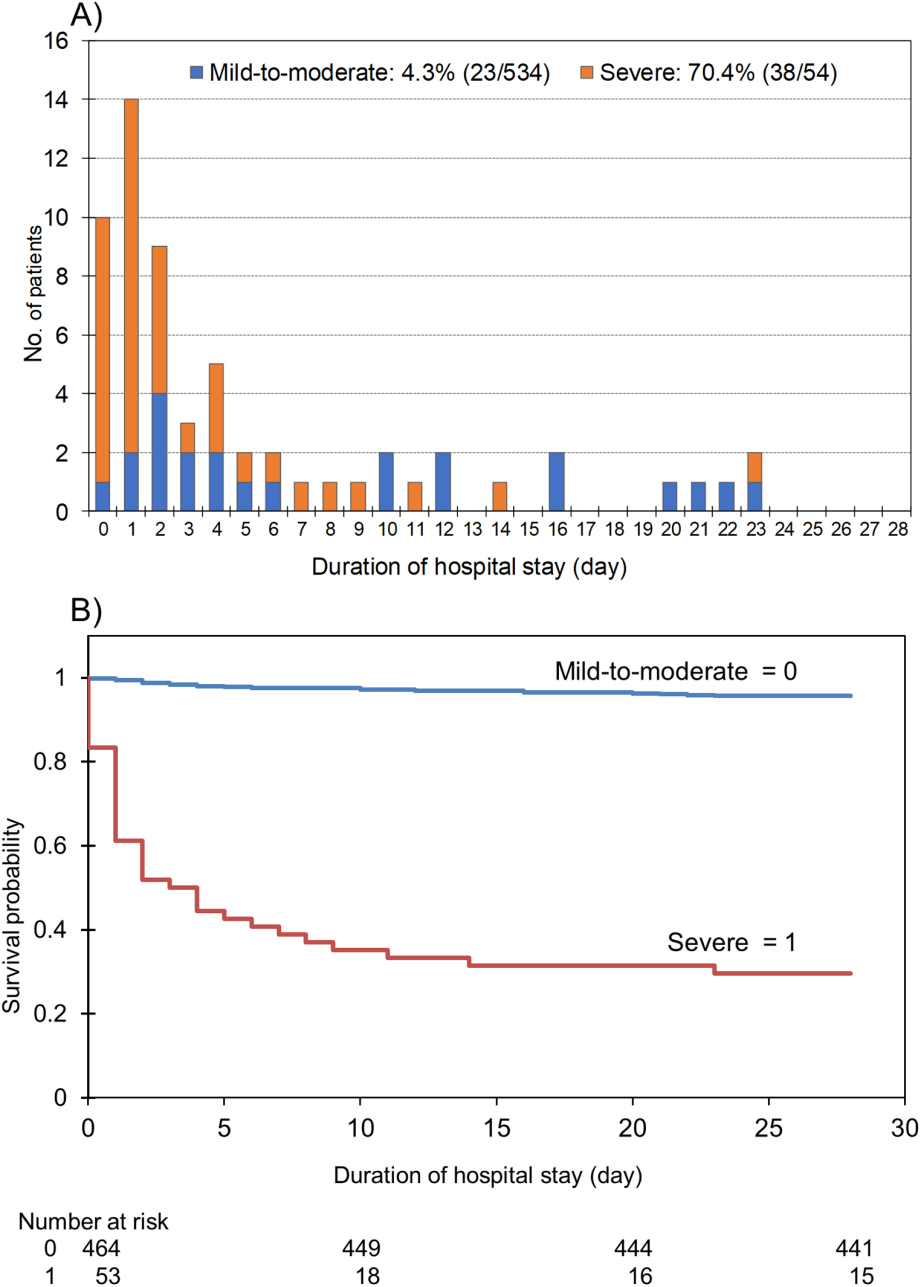
Severity and length of hospital stay in fatal cases of invasive *Streptococcus dysgalactiae* subsp. *equisimilis* infections (**a**) and Kaplan–Meier estimate of the probability of 28-day survival (**b**). The mortality rate was 70.4% (38/54) in severe cases and 4.3% (23/534) in mild-to-moderate cases

Figure 2 shows the relationship between disease severity, which correlated the most with death (Table 1) and duration of hospital stay in the fatal cases. The mortality rate was 70.4% (*n* = 38) in patients with severe disease and 4.3% (*n* = 23) in patients with mild-to-moderate disease. The mortality rate within 2 days after admission was 48.1% in patients with severe disease and 1.3% in those with mild-to-moderate disease (Fig. 2a**).** Based on Kaplan–Meier analysis, we estimated the probability of survival to 28 days of hospitalisation **(**Fig. 2b**)**. Patients with severe disease were significantly less likely to survive 28 days than those with mild-to-moderate disease (*p* < 0.001, log-rank test). The Kaplan–Meier curves indicated that death risk was the highest during the first 2 days after hospitalisation.

Kaplan–Meier analysis indicated a significant difference for the WBC count, PLT count, Cr level, and CK level between the survival and mortality groups in the univariate analysis, as shown in Supplementary Fig. 4. Abnormal levels of leukopenia (WBC < 4,000 cells/µL), thrombocytopenia (PLT < 10 × 10^4^ cells/µL), and organ injury (Cr ≥ 2.0 mg/dL and CK ≥ 300 IU/L) were associated with decreased survival (*p* < 0.001, log-rank test). Additionally, cases of leukopenia were more likely to end fatally within 5 days.

### Prognostic factors related to 28-day mortality

Risk factors associated with fatal outcomes in the multivariable analysis are shown in Table 3. The analysed characteristics were age, disease severity, clinical manifestations, biomarkers, and macrolide resistance. After excluding biomarkers that were identified as confounding factors, Cr and CK levels, which were presumed to be clinically meaningful based on the Kaplan–Meier analysis, were finally selected. Age ≥ 60 years, severe disease, bacteraemia without primary focus, other invasive infections, Cr ≥ 2.0 mg/dL, CK ≥ 300 IU/L, and macrolide resistance were significant independent risk factors for death in patients with iSDSE infections.

**Table 3:** Multivariable analysis of risk factors associated with fatal outcomes in patients with invasive *Streptococcus dysgalactiae* subsp. *equisimilis* infections

Characteristic	Survived (*n* = 456)	Died (*n* = 52)	*P*-value	HR (95% CI)
	*n* (%)	*n* (%)		
**Age groups (years)**				
18‒59	73 (16.0)	3 (5.8)	Ref	
≥ 60	383 (84.0)	49 (94.2)	**0.042**	**3.4 (1.0‒11.3)**
**Severity**				
Mild-to-Moderate	441 (96.7)	21 (40.4)	Ref	
Severe	15 (3.3)	31 (59.6)	**< 0.001**	**15.0 (7.7‒29.2)**
**Clinical manifestations**				
Cellulitis	191 (41.9)	1 (1.9)	Ref	
Bacteraemia without primary focus	147 (32.2)	31 (59.6)	**0.003**	**20.5 (2.8‒152.3)**
Others ^a^	118 (25.9)	20 (38.5)	**0.016**	**12.2 (1.6‒93.2)**
**Biomarkers**				
Creatinine (≥ 2.0 mg/dL)	58 (12.7)	21 (40.4)	**0.010**	**2.2 (1.2‒4.0)**
Creatine kinase (≥ 300 IU/L)	98 (21.5)	31 (59.6)	**0.019**	**2.1 (1.1‒3.8)**
**Macrolide susceptibility**				
Susceptible	377 (82.7)	34 (65.4)	Ref	
Resistance ^b^	79 (17.3)	18 (34.6)	**0.048**	**1.8 (1.0‒3.3)**

^a^ Includes septic arthritis, bacteraemic pneumonia, abscess, NF, osteomyelitis/spondylitis, erysipelas, endocarditis, meningitis, and STSS.

^b^ Possessing *mefA, ermA*, or *ermB* genes that mediate macrolide and clindamycin resistance

### Antibiotic susceptibility

The MIC range, MIC_50_, and MIC_90_ of ten antimicrobial agents for SDSE strains are shown in Supplementary Table 1. With the exception of cefazolin, all β-lactam agents tested presented excellent susceptibility with MIC_90_s from 0.063 to 0.031 µg/mL. No mutations were identified in *pbp1a*, *pbp2x*, and *pbp2b* encoding the respective penicillin-binding proteins associated with β-lactam resistance (data not shown).

### Association between *emm* type and patient prognosis

The proportion of the *emm* type in all isolates divided into survival and mortality groups is shown in Supplementary Fig. 5. In *stG10* and *stG5420*, the proportion of fatal cases was slightly higher, but there was no significant difference between the groups for all types, including *stG6792*, which is the most isolated in Japan.

## Discussion

In this most extensive surveillance of adults with iSDSE infections in Japan, we revealed an overall 28-day mortality rate of 10.4%, which was significantly associated with older age (≥ 60 years), disease severity, clinical manifestations, biomarkers on admission, and macrolide resistance.

As expected, disease severity on admission was an independent risk factor significantly associated with mortality. Clinical evaluation of disease severity according to the guidelines is crucial for physicians to promptly predict iSDSE infection prognosis.

Here, the number of patients and mortality rate increased with age, and advancing age had a large effect on the mortality rate. Moreover, most patients with iSDSE infections had various underlying diseases. Inflammatory responses to SDSE infection may be impaired with aging, leading to fragility and increased susceptibility to recurrent or persistent infections. Chronic inflammation associated with aging, termed ‘inflammaging’, is a risk factor for diabetes mellitus, malignancy, cardiovascular disease, and kidney disease [23]. We hypothesise that these processes, including aging, SDSE colonisation, and underlying disease progression, contribute considerably to the morbidity and mortality of patients with SDSE infections in Japan, which is a super-aged society.

Bacteraemia without primary focus and other invasive infections (such as bacteraemic pneumonia, NF, meningitis, and STSS) had poor prognosis compared to cellulitis. The fatality rates were 18.0 and 12.4%, respectively, which are consistent with those reported in previous studies, with 13 to 20% [7–9, 14, 24].

Regarding the laboratory findings, WBC < 4,000 cells/µL, Cr ≥ 2.0 mg/dL, and CK ≥ 300 IU/L were important biomarkers and correlated strongly with poor outcomes. Studies describing biomarkers as predictors of mortality in patients with SDSE are limited. One previous study reported that patients with SDSE infections had an increased mortality rate with leukopenia (WBC < 5,000 cells/µL) and thrombocytopenia (PLT < 13.0 × 10^4^ cells/µL) [13]. International guidelines [18, 20, 21] on severe conditions in patients with each disease particularly emphasise leukopenia (WBC < 4,000 cells/µL). Additionally, acute tubular necrosis resulting from sepsis-induced tissue hypotension and/or hypoxemia may contribute to renal injury, as shown by the laboratory findings such as Cr ≥ 2.0 mg/dL. Moreover, the serum concentrations of CK, which is released into the bloodstream when muscle cells disintegrate, increased in patients with poor outcomes. Thus, high CK concentrations may reflect extensive or systemic injury caused directly by SDSE infections or indirectly by hypoperfusion, indicating multiple organ damage rather than a specific diagnosis. Our study also revealed that patients infected by isolates harbouring the macrolide-resistance gene had a significantly poor prognosis. The mechanism and clinical relevance remain to be explored.

Regarding the *emm* type, *stG6792* has constantly been the most prevalent in Japan [12, 25], and it is also emerging in other countries. In European countries, *stG62647* has recently become more prevalent [13, 26, 27] in addition to *stG643*, *stG485, stG6*, and *stC74a* [10, 11, 28, 29]. According to the CDC database concerning the *emm* type, the *stG6792* reference strain appears to be derived from India, suggesting that this type was transmitted from India to Japan. The routes of transmission and the reasons for variation in the dominant *emm* types among countries should be elucidated.

Our study has some limitations. First, although we evaluated mortality risk according to the clinical manifestations based on three groups (cellulitis, bacteraemia without primary focus, and others), the risk of death for each disease might have been underestimated. For example, NF and STSS, the most serious forms of iSDSE infections, were included in the ‘others’ category, which includes various invasive diseases. However, we elucidated a poor prognosis of bacteraemia without a primary focus compared to cellulitis. Second, the number of patients with STSS, which is associated with mortality, was small (n = 4) in this study, and the proportion of STSS from SDSE is lower than that from GAS. We conducted a six-year surveillance program for the three streptococcal species GAS, SDSE, and *S. agalactiae* (GBS) as part of the “Nationwide Invasive Streptococcal Disease Surveillance”. As a result, STSS cases in adults were 7.1% (23/326) for GAS and 0.7% (4/588) for SDSE, and 0.5% (2/443) for GBS. We included patients with invasive SDSE, which was defined as isolation of SDSE from normally sterile clinical samples such as blood, cerebrospinal fluid, and closed pus. However, according to the study protocol, we excluded patients with SDSE from a wound culture accompanied by necrotizing fasciitis or streptococcal toxic shock syndrome. In addition, the unique Japanese “universal health insurance system”, which provides good access to medical institutions and makes it easy to prescribe antibiotics could have had an impact on morbidity and mortality. Moreover, the differences in lifestyle habits between Japan and western countries may have influenced the results. Future surveillance will need to elucidate this issue through global collaboration. Third, we could not analyse missing data (lack of basic information or loss on follow-up), which accounted for 14.3% (n = 98) of the total cases based on our retrospective design. However, we confirmed that the demographics, clinical manifestations, and prognosis of those patients did not significantly differ from those included in this study. Fourth, we categorised disease severity into two groups, ‘mild-to-moderate’ and ‘severe’. As shown in Table 1, various diseases comprise iSDSE infections. For example, the practice guidelines for skin and soft tissue infection [18] define disease severity as “mild,” “moderate,” and “severe,” whereas those for other diseases [20, 21] categorise severity into “non-severe” and “severe.” Therefore, we divided patients with iSDSE infections into “mild-to-moderate” and “severe” groups. Finally, the proportion of antibiotic classes as the initial therapy differed between the survival and mortality groups because the empiric antibiotic treatment was influenced by apparent disease severity. Furthermore, combination therapy of penicillin with clindamycin for life-threatening infections was given to a small number of patients, and its efficacy remains unclear.

In conclusion, our results provide evidence of prognostic factors for death due to iSDSE infections and the basis for evaluating clinical manifestations, disease severity, and biomarkers on admission. The prevalence and burden of iSDSE infection, which particularly affects older adults with underlying diseases, are likely to further increase in developed countries. Strategies to reduce the mortality risk of iSDSE infections require further investigations of dynamic molecular epidemiology and clinical characteristics, as well as optimal treatment based on well-conducted studies.

### Electronic supplementary material

Below is the link to the electronic supplementary material.

Supplementary Material 1

Supplementary Material 2

Supplementary Material 3

Supplementary Material 4

Supplementary Material 5

Supplementary Material 6

## Data Availability

No datasets were generated or analysed during the current study.
