# Supplementary figures and images for: Clinical manifestations and biomarkers to predict mortality risk in adults with invasive Streptococcus dysgalactiae subsp. equisimilis infections
Source: Eur J Clin Microbiol Infect Dis. 2024 Jun 7;43(8):1609–19. doi: 10.1007/s10096-024-04861-4 (PMC11271329; doi:10.1007/s10096-024-04861-4)

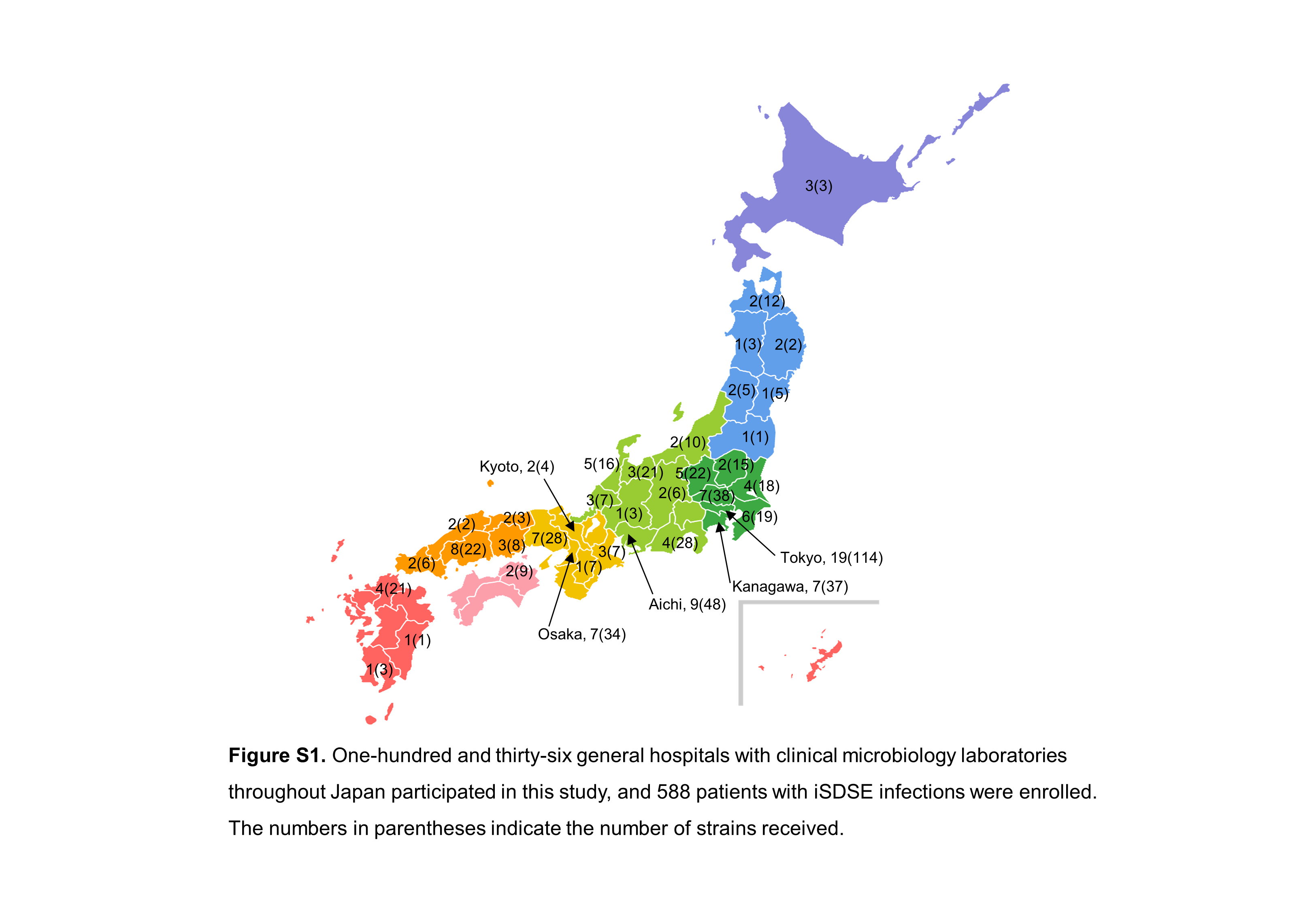

Supplement: Supplementary file 1 — Supplementary Material 1 [file 10096_2024_4861_MOESM1_ESM.tif]

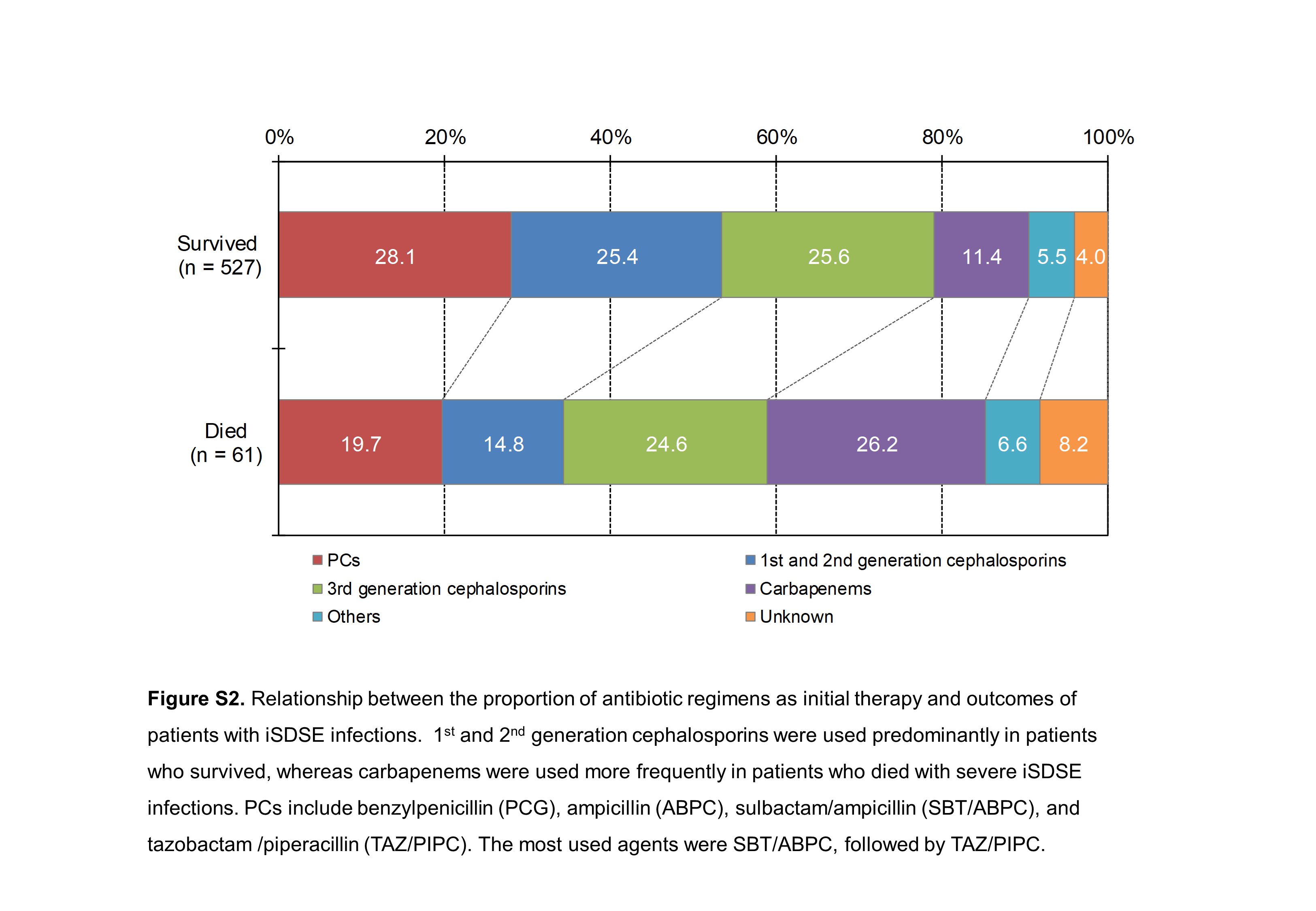

Supplement: Supplementary file 2 — Supplementary Material 2 [file 10096_2024_4861_MOESM2_ESM.tif]

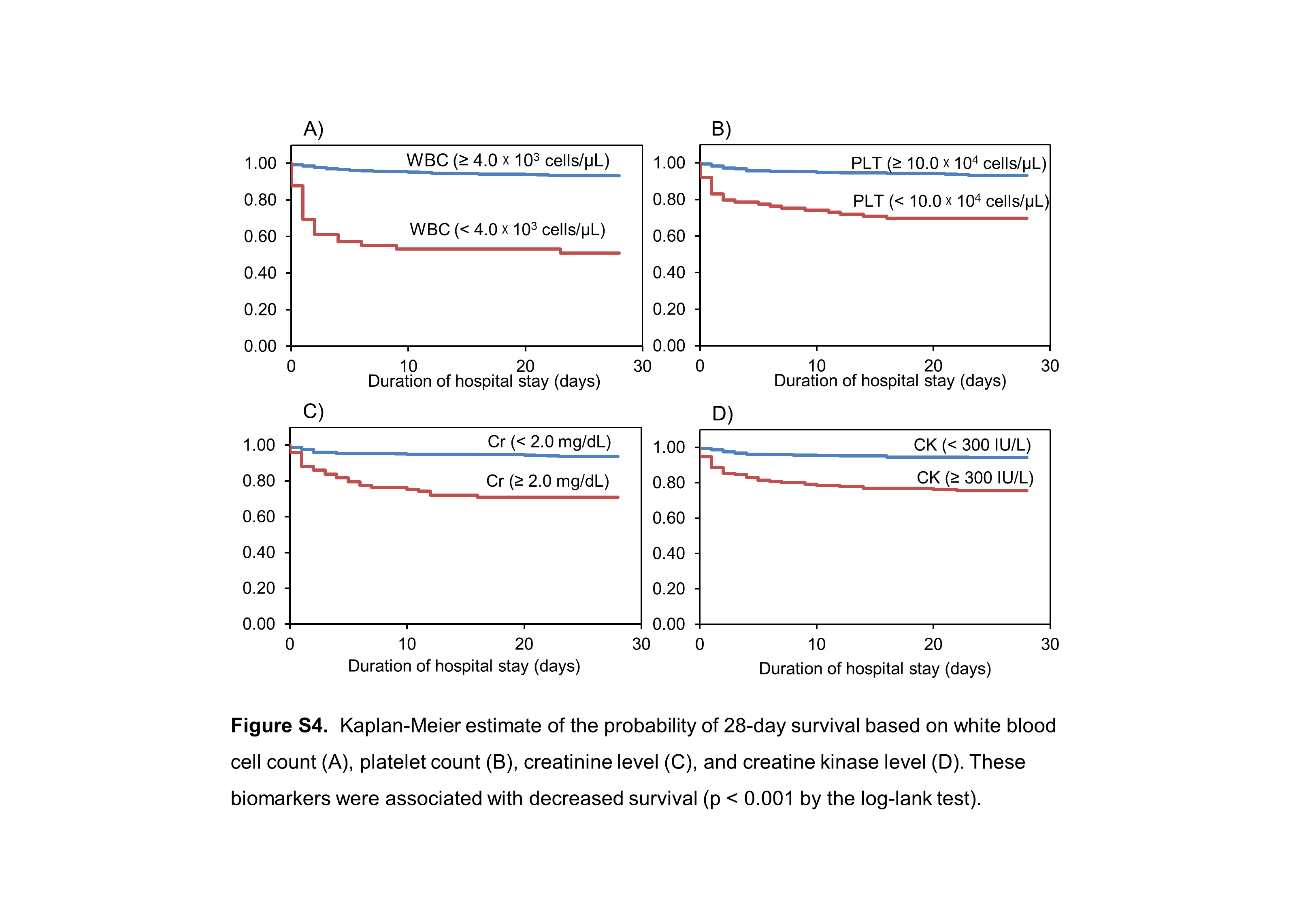

Supplement: Supplementary file 3 — Supplementary Material 3 [file 10096_2024_4861_MOESM3_ESM.tif]

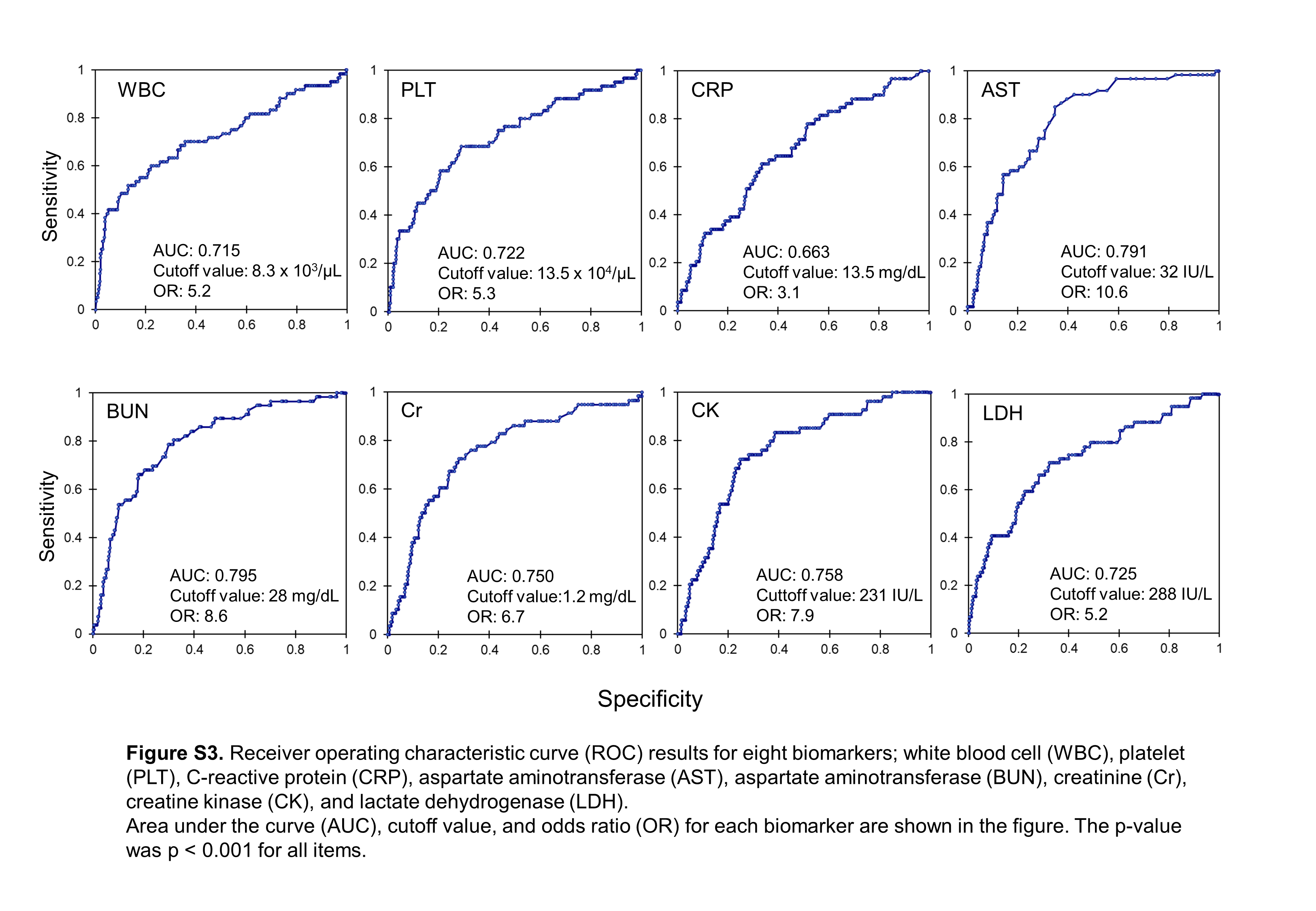

Supplement: Supplementary file 4 — Supplementary Material 4 [file 10096_2024_4861_MOESM4_ESM.tif]

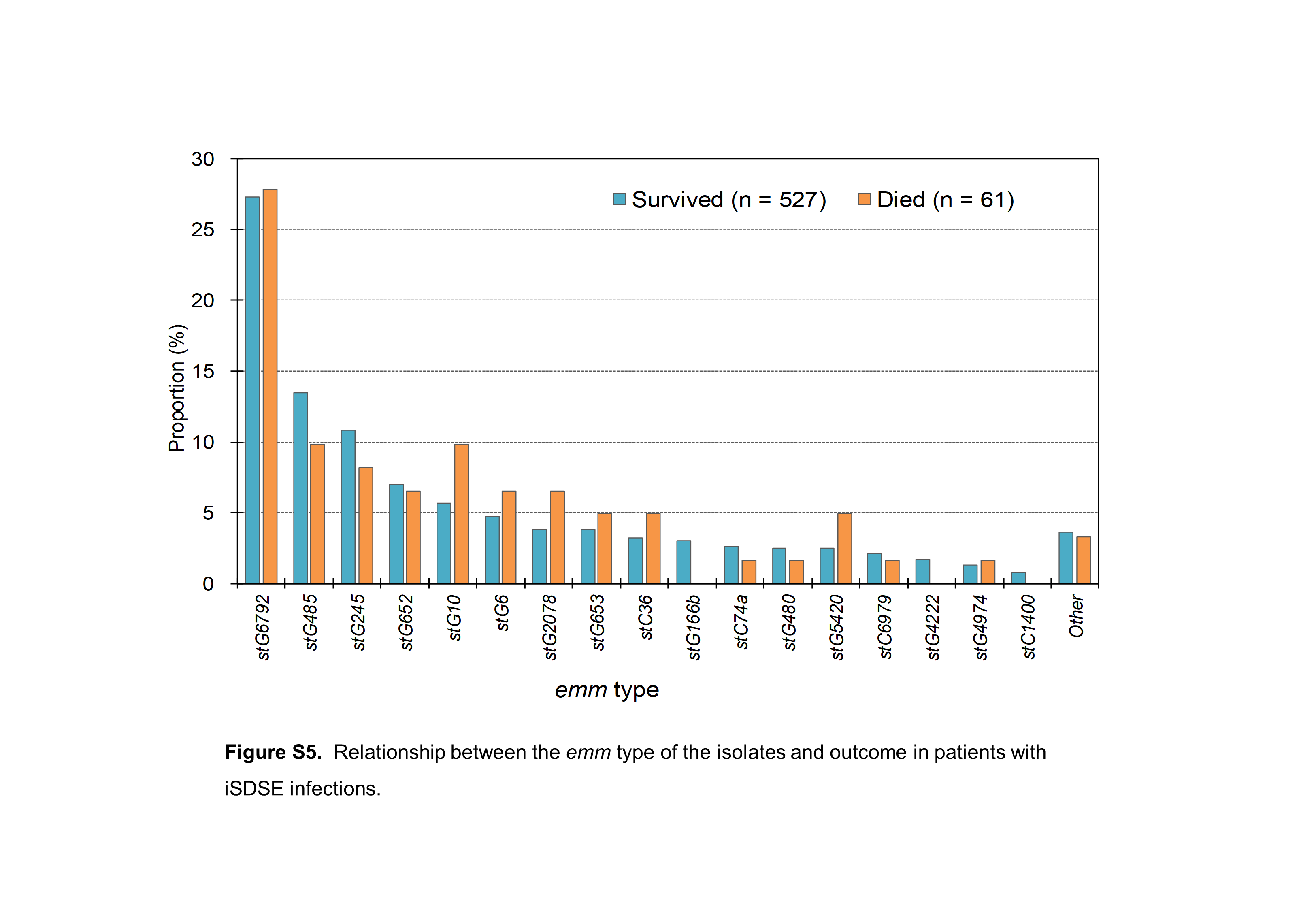

Supplement: Supplementary file 5 — Supplementary Material 5 [file 10096_2024_4861_MOESM5_ESM.tif]
