# Supplementary material for: Clinical manifestations and biomarkers to predict mortality risk in adults with invasive Streptococcus dysgalactiae subsp. equisimilis infections
Source: Eur J Clin Microbiol Infect Dis. 2024 Jun 7;43(8):1609–19. doi: 10.1007/s10096-024-04861-4 (PMC11271329; doi:10.1007/s10096-024-04861-4)
Supplement: Supplementary file 6 — Supplementary Material 6 [file 10096_2024_4861_MOESM6_ESM.docx]

**Supplementary File**

**Clinical Manifestations and Biomarkers to Predict Mortality Risk in Adults with Invasive *Streptococcus dysgalactiae* subsp. *equisimilis* Infections**

Shigeo Hanada^1,2,3^*, Takeaki Wajima^4^, Misako Takata^3^, Miyuki Morozumi^3^, Michi Shoji^5^, Satoshi Iwata^3^, Kimiko Ubukata^3,6^*

**Affiliations:**

^1)^ Department of Respiratory Medicine, Respiratory Center, Toranomon Hospital, Tokyo, Japan

^2)^ Okinaka Memorial Institute for Medical Research, Tokyo, Japan

^3)^ Department of Microbiology, Tokyo Medical University, Tokyo, Japan

^4)^ Department of Microbiology, Faculty of Pharmacy, Meijo University, Nagoya, Japan

^5)^ Department of Infectious Disease, National Sanatorium Tamazenshoen, Tokyo Japan

^6)^ Center for General Medicine Education, Keio University School of Medicine, Tokyo, Japan

**Corresponding Author**:

Shigeo Hanada, M.D., Ph.D.

Department of Respiratory Medicine, Respiratory Center, Toranomon Hospital, 2-2-2 Toranomon, Minato-ku, Tokyo 105-8470, Japan

E-mail: [gourouhanada@yahoo.co.jp](mailto:gourouhanada@yahoo.co.jp)

Fax: +81-3-3582-7068; Phone: +81-3-3588-1111

**Table S1**. MIC range, MIC_50_, and MIC_90_ of 10 antimicrobial agents against *Streptococcus* *dysgalactiae* subsp. *equisimilis* collected in this study (n = 588).

| Antimicrobial agent | MIC range (µg/mL) | MIC_50_ (µg/mL)^a^ | MIC_90_ (µg/mL)^b^ |
| --- | --- | --- | --- |
| ***Oral*** |  |  |  |
| Penicillin G | 0.008–0.016 | 0.016 | 0.016 |
| Ampicillin | 0.016–0.031 | 0.031 | 0.031 |
| Amoxicillin | 0.016–0.031 | 0.031 | 0.031 |
| Cefdinir | 0.008–0.031 | 0.016 | 0.016 |
| Cefditoren | 0.008–0.016 | 0.016 | 0.016 |
| ***Parenteral*** |  |  |  |
| Cefazolin | 0.063–0.25 | 0.125 | 0.125 |
| Cefotaxime | 0.008–0.031 | 0.016 | 0.016 |
| Ceftriaxone | 0.008–0.031 | 0.016 | 0.016 |
| Meropenem | 0.008–0.016 | 0.016 | 0.016 |
| Vancomycin | 0.25–1 | 0.5 | 0.5 |

^a^ MIC_50_, minimum inhibitory concentration required to inhibit 50% of isolates;

^b^ MIC_90_, minimum inhibitory concentration required to inhibit 90% of isolates.

Among the isolates, no strains with abnormal *pbp1a*, *pbp2x*, and *pbp2b* associated with β-lactam antibiotic resistance were identified.
